# Review: The Role of Wnt/β-Catenin Signalling in Neural Crest Development in Zebrafish

**DOI:** 10.3389/fcell.2021.782445

**Published:** 2021-11-29

**Authors:** Gemma Sutton, Robert N. Kelsh, Steffen Scholpp

**Affiliations:** ^1^ Living Systems Institute, School of Biosciences, College of Life and Environmental Sciences, University of Exeter, Exeter, United Kingdom; ^2^ Department of Biology and Biochemistry, University of Bath, Bath, United Kingdom

**Keywords:** Wnt/β-catenin signalling, neural crest, gene regulatory network, NC induction, NC specification, pigment cells, melanocyte, Zebrafish

## Abstract

The neural crest (NC) is a multipotent cell population in vertebrate embryos with extraordinary migratory capacity. The NC is crucial for vertebrate development and forms a myriad of cell derivatives throughout the body, including pigment cells, neuronal cells of the peripheral nervous system, cardiomyocytes and skeletogenic cells in craniofacial tissue. NC induction occurs at the end of gastrulation when the multipotent population of NC progenitors emerges in the ectodermal germ layer in the neural plate border region. In the process of NC fate specification, fate-specific markers are expressed in multipotent progenitors, which subsequently adopt a specific fate. Thus, NC cells delaminate from the neural plate border and migrate extensively throughout the embryo until they differentiate into various cell derivatives. Multiple signalling pathways regulate the processes of NC induction and specification. This review explores the ongoing role of the Wnt/β-catenin signalling pathway during NC development, focusing on research undertaken in the Teleost model organism, zebrafish (*Danio rerio*). We discuss the function of the Wnt/β-catenin signalling pathway in inducing the NC within the neural plate border and the specification of melanocytes from the NC. The current understanding of NC development suggests a continual role of Wnt/β-catenin signalling in activating and maintaining the gene regulatory network during NC induction and pigment cell specification. We relate this to emerging models and hypotheses on NC fate restriction. Finally, we highlight the ongoing challenges facing NC research, current gaps in knowledge, and this field’s potential future directions.

## Introduction

The neural crest (NC) is a transient and multipotent embryonic cell population with extraordinary migratory capacity. The NC is crucial for vertebrate development and is an entire model system in its own right in developmental biology. NC cells (NCCs) give rise to many cell derivatives, including body pigment cells, neuronal cells of the peripheral nervous system, cardiomyocytes, and skeletogenic cells in craniofacial tissue (Le Douarin, 1999). Although recently an NC rudiment was identified in tunicates (Abitua et al., 2012), the NC is considered as an evolutionary novelty of vertebrates that facilitated the emergence of the specialised vertebrate cranium, including the hinged jaw, specialised neural structures and sensory organs (Gans and Northcutt, 1983). This emergence of the NC population and the “new head” enabled vertebrates to acquire active feeding behaviours and contributed to the remarkable radiation of the vertebrate lineage (Hall, 2000).

**FIGURE 1:**
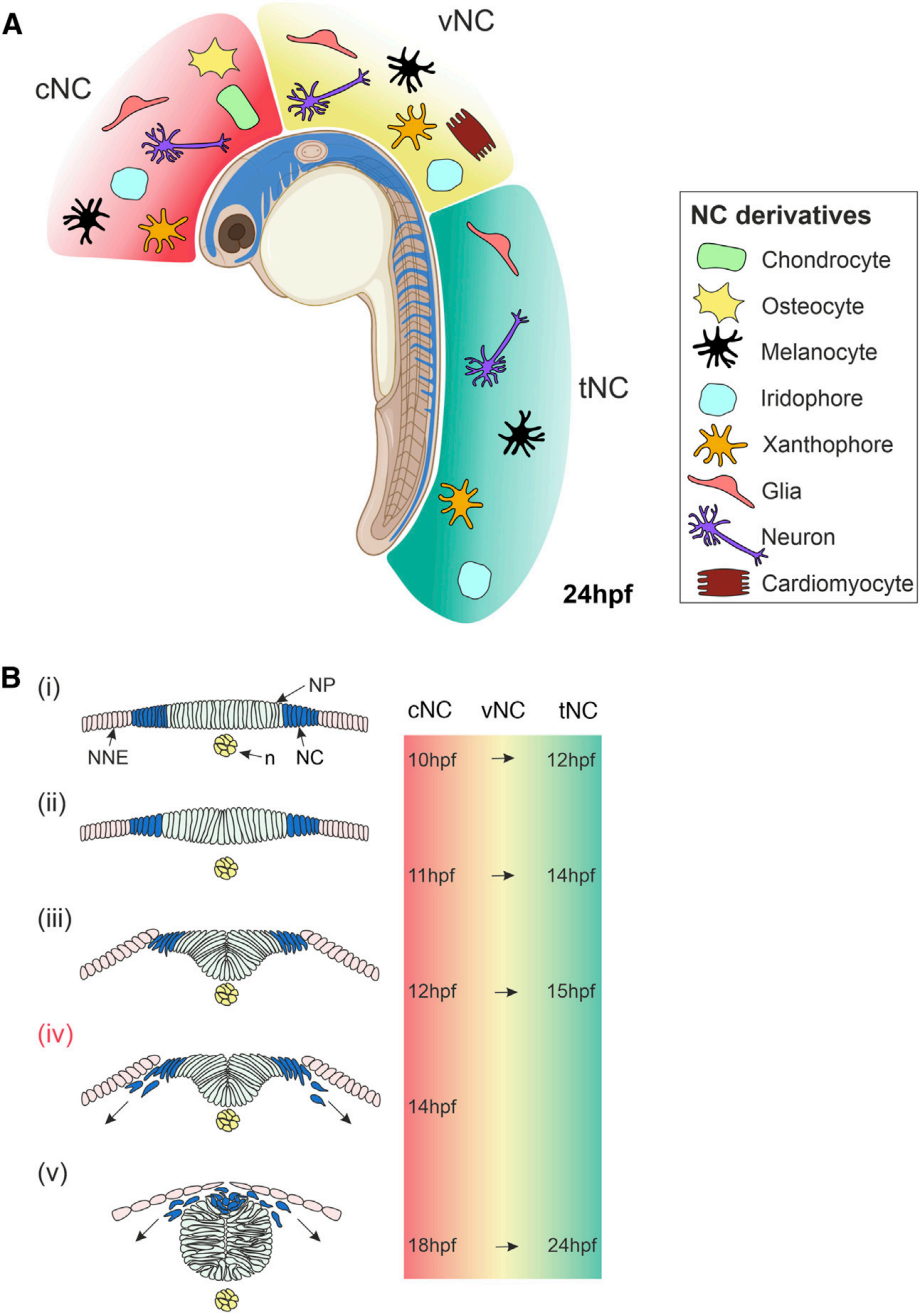
Overview of Neural Crest Development in Zebrafish. **(A)** Lateral view of a zebrafish embryo at 24 hpf showing NC cells (blue). The NC is subdivided along the anteroposterior axis into cNC, vNC and tNC, indicated in red, yellow and green, respectively. These NC populations give rise to different NC derivatives. **(B)** Cross-sections of zebrafish embryo showing NC cells emerge, delaminate and start migrating. **(i)** NC cells originate in the neural plate border at the interface of the NNE and NP. **(ii,iii)** NC cells begin to delaminate during the formation of the neural keel. **(iv)** Cranial NC cells begin migrating as the neural keel folds up (Schilling and Kimmel, 1994). **(v)** When the neural rod has formed., NC cells emerge from the neural plate border and the dorsal neural tube, delaminate and begin migrating around the embryo. The times at which these stages of NC development occur in zebrafish are indicated on the right for cNC at the level of the midbrain and for tNC at the level of the somites. The time of onset of vNC migration is yet to be defined. Schematics of transverse sections of zebrafish embryos indicate dorsal to the top **(Bi–v)**. cNC, cranial neural crest; vNC, vagal neural crest; tNC, trunk neural crest; NC, neural crest; NP, neural plate; NNE, non-neural ectoderm; n, notochord; hpf, hours post-fertilisation. Created with BioRender.com.

NC development begins during early embryogenesis, specifically during gastrulation, in the ectodermal germ layer and proceeds concurrently with neurulation (Figure 1). The NC is induced within a region of ectoderm located at the non-neural and neural ectoderm interface, known as the neural plate border (NPB). NCCs undergo epithelial-to-mesenchymal transition (EMT) in an anterior-posterior (AP) progression and migrate large distances across the embryo, during which they integrate a wide array of signals and execute a complex choreography of gene regulatory changes until their final cell fates at their final destinations in the body are determined. The timing of the specification of multipotent NCC progenitors towards their final cellular fate is only partially defined; for the better-studied melanocytes, it is thought to initiate prior to migration.

Developmental biologists have used various vertebrate model organisms to gain insight into the fundamental biology of the NC, including zebrafish, amphibians, chicks and mice. In the mid-20th century, the quail-chick chimaera system advanced NC research, uncovering specific migratory pathways and cell fates (Le Douarin, 1973). In these fundamental fate-mapping studies, homotopic and heterotopic transplantation of quail NCCs to chick embryos enabled the regional fate mapping of NCCs *in vivo* and established NC plasticity (Le Douarin, 1999). At the end of the 20th century, similar fate-mapping experiments were carried out in zebrafish by microinjecting fluorescent dyes and imaging by light and electron microscopy (Raible et al., 1992; Schilling and Kimmel, 1994). These studies demonstrated the extraordinary potency of the NC and revealed the conservation of NCC derivatives and migratory pathways in zebrafish and other vertebrates. Subsequently, one study extended these iontophoretic labelling investigations, confirming both the apparent fate-restriction of many NCCs and the small size of wild-type zebrafish NCC clones. It also characterised a partial failure of NC migration and fate specification in the zebrafish mutants of Sox10 transcription factor (Dutton et al., 2001).

The NC fate map demonstrated discrete populations of NCCs along the AP axis, which are conserved across the vertebrate models investigated. Cranial NC is the anterior-most NC population that emerges from the NPB, adjacent to the midbrain and hindbrain position of the developing embryo (Kague et al., 2012). Cranial NC derivatives contribute to the craniofacial skeleton and gills, as well as neurons of the sensory and parasympathetic ganglia and pigment cells (Figure 1A) (Kague et al., 2012; Lee et al., 2013; Mongera et al., 2013; Schilling and Kimmel, 1994). Trunk NC cells are located more posteriorly, originating along the spinal cord, adjacent to the somites. Trunk NC forms neuronal and glial derivatives in the dorsal root ganglia (DRG), sympathetic and parasympathetic ganglia, and pigment cells (Figure 1A) (An et al., 2002; Raible and Eisen, 1994; Raible et al., 1992). The cardiac/vagal NC domain is located between the trunk and cranial NC. This neural crest population spans from immediately rostral to the otic vesicle to caudal to somite 6 (Sato and Yost, 2003). The precise AP boundaries of the zebrafish cardiac/vagal NC domain remain to be fully defined. However, cells from this NC group are essential for zebrafish heart looping and contribute neurons and glia to the enteric nervous system (Figure 1A) (Shepherd et al., 2004; Elworthy et al., 2005; Olden et al., 2008). A recent study suggests that two distinct populations of cardiac/vagal NC migrate to the heart, with one population forming cardiomyocytes in the heart tube and the other migrating to the bulbus arteriosus (Cavanaugh et al., 2015). Pigment cells are a ubiquitous feature of the NC and are not regionally localised. Paracrine signalling from surrounding cells is essential for the specification of these various NC derivatives; however, many aspects of this communication process and its implication in fate restriction is not well understood.

During vertebrate development, the Wnt/β-catenin signalling pathway acts as a long-range morphogen system providing concentration-dependent positional information in many tissues and organs. Moreover, many experimental observations from multiple model systems suggested that the Wnt/β-catenin signalling pathway is essential for the emergence of the NC. For example, in *Xenopus* animal cap explants, over-expression of Wnt ligands induces expression of NC markers, whereas expression of dominant-negative Wnts represses the expression of these markers (SaintJeannet et al., 1997; Chang and Hemmati-Brivanlou, 1998; LaBonne and Bronner-Fraser, 1998; Bang et al., 1999; Tan et al., 2001; Villanueva et al., 2002). Similarly, in avian embryos, inhibition of Wnt/β-catenin signalling blocks NC marker expression and the addition of Wnt to neural tube explants is sufficient to induce NC (Garcia-Castro et al., 2002). Furthermore, exogenous Wnt/β-catenin signalling can induce NC in human inducible pluripotent stem cells (Gomez et al., 2019).

There is also extensive evidence supporting the requirement of Wnt/β-catenin signalling after forming the NC during the selection of differentiation pathways, reversible fate specification followed by final, irreversible fate determination. For example, in mice, Wnt/β-catenin signalling promotes the specification of sensory neurons and melanocytes over alternative NC fates (Hari et al., 2002; Lee et al., 2004). Furthermore, in zebrafish, activation of Wnt/β-catenin signalling in the pre-migratory NC promotes pigment cell types at the expense of neuronal cell fates (Dorsky et al., 1998). These findings have led to the perception that the Wnt/β-catenin signalling pathway is required in two stages of NC development, firstly in NC induction and secondly in the specification of NC derivatives.

This review will primarily discuss NC induction and specification processes, with a focus on pigment cell determination. We will provide an extensive analysis of the role of the Wnt/β-catenin signalling pathway during these stages of NC development. We will highlight contributions from studies undertaken in the Teleost model organism, zebrafish, and compare and contrast these to findings from other vertebrate model systems. Although zebrafish was a relatively late arrival to NC research compared to other model organisms, we believe this model excels in studies of NC development, particularly in fate specification. Zebrafish is now established at the forefront of NC research due to the powerful genetic and transgenic tools available and transparent embryos that make ideal samples for high- and super-resolution imaging. Finally, we will discuss the potential future directions of Wnt/β-catenin signalling research in the context of the NC and anticipate the upcoming challenges of this field.

### The Wnt/β-Catenin Signalling Pathway

Wnt proteins are secreted ligands that activate signalling pathways that regulate many developmental processes (Angers and Moon, 2009; Nusse and Clevers, 2017). Wnt signalling controls pattern formation and cell behaviour through changes in gene expression and cell morphology. Wnt signal transduction pathways are classified as β-catenin dependent (also known as the canonical pathway) or β-catenin independent (the non-canonical pathways). The type of Wnt signalling pathway activated depends on the combination of Wnt ligands, receptors and co-receptors, and the cellular context (Niehrs, 2012). There is significant variation in the number of Wnt genes between different species. For example, 19 Wnt genes have been identified in humans and mice, whereas zebrafish can have up to 25 Wnt genes due to the teleost-specific whole-genome duplication (Miller, 2002; Duncan et al., 2015; Ruzicka et al., 2019). There are also a variety of Wnt receptors and co-receptors. The seven-pass-membrane protein Frizzled is the predominant receptor of Wnt signalling pathways. Mammals have 10 Frizzled genes, and zebrafish are predicted to have at least 17 Frizzled genes (Nikaido et al., 2013; Ruzicka et al., 2019).

**FIGURE 2:**
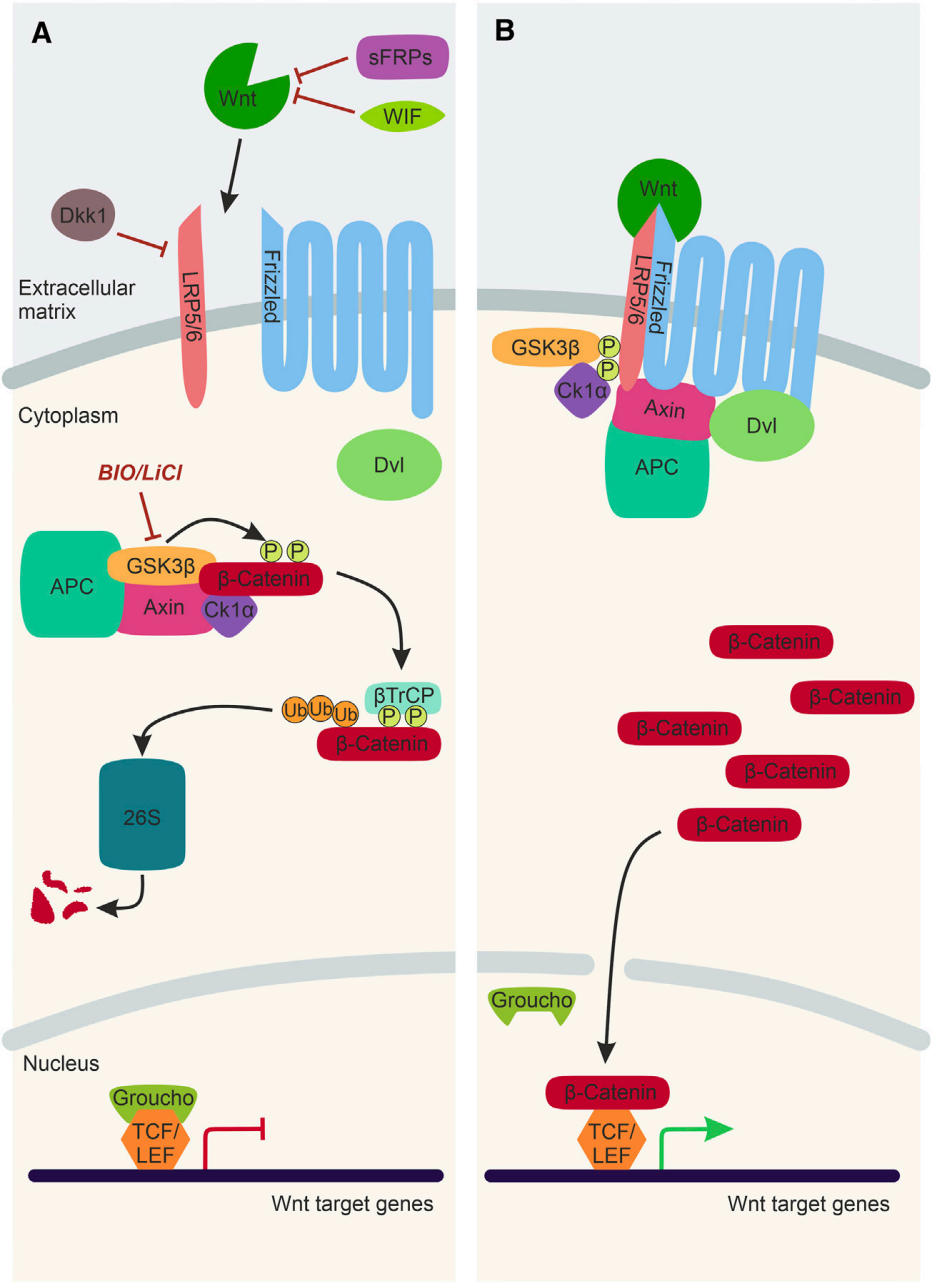
The Wnt/β-catenin Signalling Pathway. **(A)** In the absence of a Wnt ligand, transcription co-activator β-catenin is phosphorylated by the destruction complex, composed of Gsk3β, Axin, CK1α and APC. Following phosphorylation, β-catenin is ubiquitinated by βTrCP and sent to the 26S proteasome for degradation. Thus, Wnt target genes, which are repressed by TCF/LEF factors and Groucho, cannot be expressed. Activation of the Wnt/β-catenin signalling pathway can be inhibited by WIF and sFRPs and Dkk1. LiCl and BIO are chemical inhibitors of Gsk3β, which can be used experimentally to stimulate Wnt/β-catenin signalling. **(B)** In the presence of a Wnt ligand, the Wnt binds to Frizzled and Lrp5/6 co-receptors. This sequesters Dvl, Axin and the destruction complex to the plasma membrane. As a result, the destruction complex is inactivated, and β-catenin is no longer degraded and can translocate to the nucleus, bind to TCF/Lef factors and activate Wnt target gene expression. Lrp5/6, lipoprotein receptor-related protein-5/6; WIF, Wnt-inhibitory factor; sFRP, secreted Frizzled-related protein; Dkk1, Dickkopf1; Dvl, Dishevelled; Gsk3β, Glycogen synthase kinase 3β; APC, Adenomatous polyposis coli; CK1α, Casein kinase 1α; βTrCP, ubiquitin ligase; 26S, 26S proteasome; TCF/LEF, T cell factor/lymphoid enhancer factor.

Wnt/β-catenin signalling is the best characterised Wnt signalling pathway implicated in NC development (Figure 2). Canonical Wnt ligands act as morphogens by establishing a gradient and influencing receiving-cell behaviour in a concentration-dependent manner. Wnt1, Wnt3a, and Wnt8a ligands predominantly activate Wnt/β-catenin signalling, ultimately stabilising the transcription factor β-catenin that translocates to the nucleus and activates target gene expression. In the absence of a Wnt/β-catenin ligand, Axin, APC, CK1, and Gsk3β form a destruction complex in the cytoplasm that phosphorylates β-catenin resulting in proteasomal degradation of this transcriptional co-factor (Figure 2A). The Wnt/β-catenin signalling pathway is activated upon the Wnt ligand interacting with a Frizzled receptor and Lrp5/6 co-receptor. The intracellular scaffolding proteins Dishevelled (Dvl) and Axin are recruited to this membrane-tethered complex, where they interact with Frizzled and Lrp5/6, respectively. The subsequent recruitment of the destruction complex to the plasma membrane leads to its inactivation, and thus β-catenin can no longer be degraded. The stabilised β-catenin thus translocates to the nucleus, where it interacts with TCF/LEF co-transcription factors to activate Wnt target gene expression (Figure 2B).

Furthermore, there are negative regulators of the Wnt/β-catenin signalling pathway that inhibit transcription activation by β-catenin. There are secreted Wnt antagonists, including secreted Frizzled-related proteins (sFRPs) and Wnt inhibitory factors (WIF), which directly bind and sequester Wnt ligands (Hsieh et al., 1999; Leyns et al., 1997) (Figure 2A). Dickkopf1 (Dkk1) is another secreted Wnt inhibitor that interacts with the Lrp5/6 co-receptors, inhibiting the formation of the ligand/receptor/co-receptor complex required for pathway activation (Bafico et al., 2001) (Figure 2A). Wnt/β-catenin signalling can also be manipulated experimentally using chemical inhibitors such as LiCl and Bromoindirubin-3′-oxime (BIO) (Meijer et al., 2003; Alexander et al., 2014; Vibert et al., 2017). These chemical inhibitors target Gsk3β and prevent the phosphorylation and subsequent degradation of β-catenin, resulting in over-activation of the Wnt/β-catenin pathway (Figure 2A).

**FIGURE 3:**
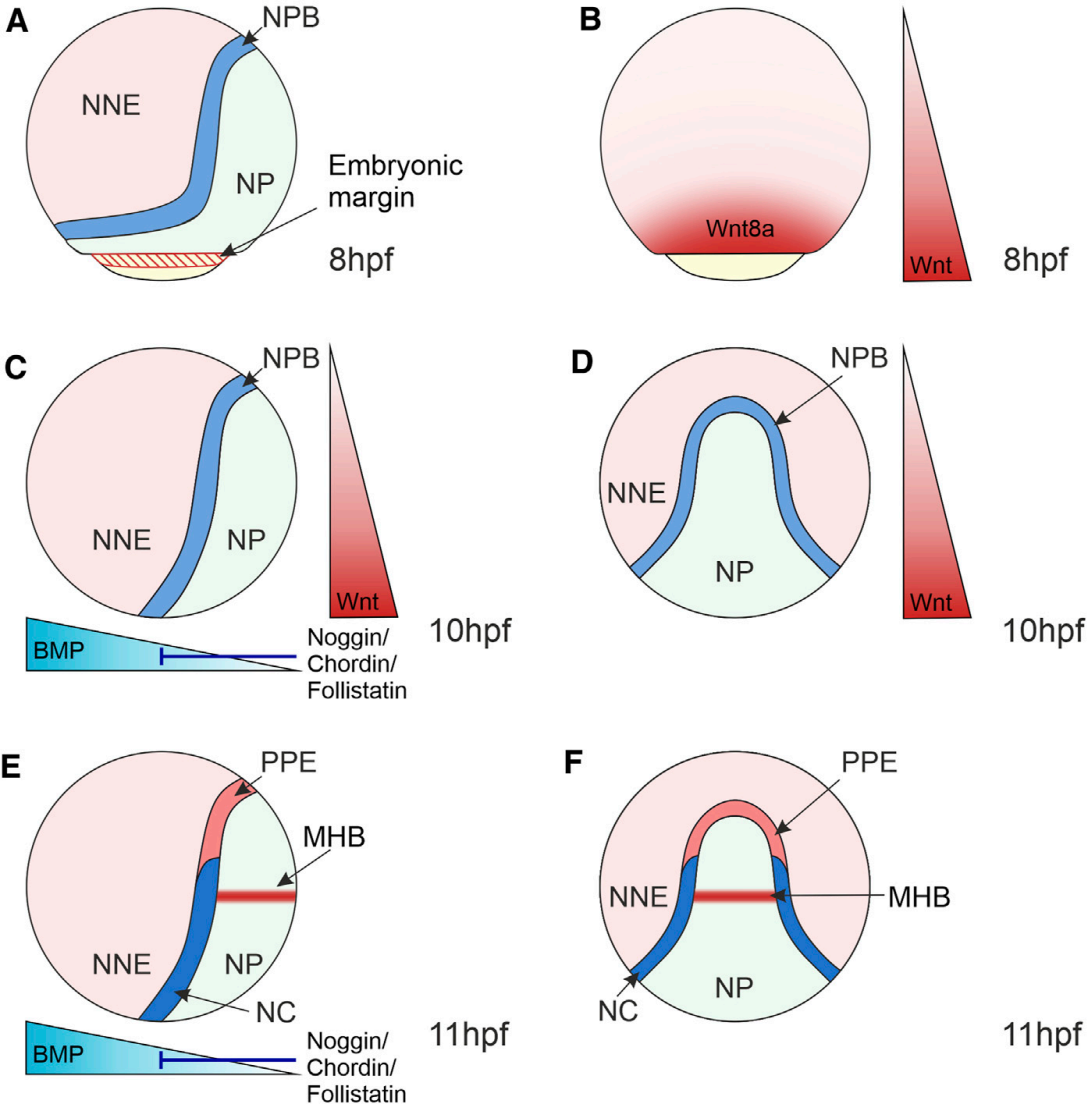
Morphogen Gradients during Neural Crest Induction in Zebrafish 8–11 hpf. **(A)** At the end of gastrulation, the ectoderm is regionalised into the NP located medially, NNE located laterally and the NPB at the interface of NNE and NP. NP and NNE will form the central nervous system and epidermis, respectively. **(B)** At 8 hpf, the Wnt ligand, Wnt8a, is expressed in a broad domain at the embryonic margin overlapping with the NPB, implicating this ligand in NC induction. **(C–F)** At the bud stage (10 hpf) and 3-somite stage (11 hpf), the dorsoventral BMP gradient is established that patterns the ectoderm, with BMPs expressed ventrally and BMP antagonists (Noggin, Chordin and Follistatin) expressed dorsally. Intermediate levels of BMP signalling induce the NPB and NC. **(C,D)** Wnt/β-catenin signalling ligands are expressed posteriorly and act to posteriorize the NP and induce NC. **(E,F)** By 11hpf a separate Wnt source is established in the MHB within the NP that is required for NC fate specification. Lateral views show anterior to the top and dorsal to the right **(A,–C,E)**. Dorsal views indicate anterior to the top **(D,F)**. NNE, non-neural ectoderm; NP, neural plate; NPB, neural plate border; NC, neural crest; PPE, pre-placodal ectoderm; MHB, midbrain-hindbrain boundary; hpf, hours post-fertilisation.

Wnt-mediated regulation of target gene expression is crucial during cell fate determination in development. During gastrulation, Wnt/β-catenin signalling is essential for the specification of posterior and ventral fates resulting from the expression of Wnt8a at the ventrolateral margin (Dorsky et al., 2002; Erter et al., 2001) (Figures 3A,B). In the ectoderm, expression of Wnt8a is crucial for the specification of posterior neural fates, but also in the induction of the NC (Lekven et al., 2001; Lewis et al., 2004). In NC development, TCF/LEF-regulated transcription plays a pivotal role in activating a gene regulatory network in the NC and subsequent NC fate restriction.

## Neural Crest Induction

NC induction takes place during gastrulation in the NPB. The NPB is exposed to signals from surrounding tissues, including the neural and non-neural ectoderm, as well as the underlying mesoderm, which induce and maintain the expression of NC markers (Figure 3). The role of signals originating from the mesoderm in NC induction was previously discounted as disruption of mesoderm involution in zebrafish embryos did not affect NC induction (Ragland and Raible, 2004). However, *Xenopus* explant experiments that conjugated animal caps with mesoderm regions found that mesoderm from the dorsolateral marginal zone specifically induced NC marker expression (Steventon et al., 2009). This suggests that mesoderm acts as a signalling source to the NC, even when involution is disrupted during gastrulation. It is essential to recognise that the NC emerges alongside other cell lineages within the NPB. For example, in the anterior NPB, the cranial NC is adjacent to the pre-placodal ectoderm that forms sensory structures in the vertebrate head and the lateral line system in aquatic vertebrates (Theveneau et al., 2013) (Figures 3E,F). In anamniote vertebrates, such as fish and amphibians, the posterior NPB gives rise to trunk NC and a population of transient embryonic sensory neurons known as Rohon-Beard cells (Lamborghini, 1980; Cornell and Eisen, 2000).

## Neural Crest Gene Regulatory Network

The induction of the NPB and NC involves the interplay of the Wnt/β-catenin signalling, Bone Morphogenetic Protein (BMP), and Fibroblast Growth Factor (FGF) pathways, which activate a complex network of transcription factors within the NC, known as the NC gene regulatory network (GRN) (Sauka-Spengler and Bronner-Fraser, 2008). The NC GRN in zebrafish has been structured as a hierarchy of transcription factors. Here we provide a brief overview of the zebrafish NC GRN; a more detailed analysis can be found in a recent review (Rocha et al., 2020).

A combination of extracellular signals activates the NC GRN. During gastrulation, the first group of transcription factors activated are coined “NPB specifiers”. At the end of gastrulation, the NC is specified within the NPB, a process characterised by the expression of further transcription factors, collectively referred to as “NC specifiers”. These NC specifiers are activated by the upstream NPB specifiers and from extracellular signalling inputs. The action of the NC specifiers, combined with extracellular signals, subsequently activates lineage-specific GRNs in the process of NC specification and subsequent fate commitment (see section on NC specification).

## Wnt Signalling and the Neural Crest Gene Regulatory Network

Wnt/β-catenin signalling, as well as BMP and FGF signalling pathways, coordinate the activation of the NC GRN. A BMP morphogen gradient is established through the expression of BMPs in the ventral side of the embryo and BMP antagonists, Noggin, Chordin and Follistatin, from the dorsal side (Hammerschmidt et al., 1996) (Figures 3C,E). This gradient patterns the ectoderm along the dorsoventral axis, with those cells that receive intermediate BMP levels forming the NC (Nguyen et al., 1998; Tucker et al., 2008; Schumacher et al., 2011).

Wnt/β-catenin signalling plays a fundamental role during zebrafish NC induction. In zebrafish, this was elucidated using a conditional heatshock promotor that activates expression of a mutant version of the TCF co-transcription factor that cannot bind β-catenin and thus blocks target gene expression (Figure 2). Expression of this mutant TCF during gastrulation resulted in a loss of expression of the NC specifier gene *foxd3* (Lewis et al., 2004). Interestingly, this approach did not affect the expression of markers of the neighbouring Rohon-Beard cells, suggesting a specific requirement of Wnt/β-catenin signalling in NC induction. In this study, Wnt8a was implicated in NC induction due to localised expression in the presumptive NC domain that overlapped with the expression of *pax3* NPB specifier during gastrulation (Figures 3A,B). Furthermore, the Morpholino (MO)-based knockdown of Wnt8a resulted in the loss of expression of the NPB specifier *pax3* and the NC specifiers *sox10* and *foxd3*. MO oligomers were microinjected at the 1-cell stage of zebrafish development. Therefore, it is not clear whether changes in NC specifier expression domains in the MO knockdowns reflects a role of Wnt8a specifically during NC induction or a downstream effect resulting from defects in Wnt8a signalling in early embryo development (Lewis et al., 2004). However, TCF/LEF binding sites have been identified in the *sox10* promoter, suggesting that this NC specifier is directly regulated by Wnt/β-catenin signalling (Dutton et al., 2008).

A further study on NC induction used a heat-shock promoter to overexpress the Wnt antagonist Dickkopf1 (Dkk1) that inhibits the Wnt/β-catenin signalling pathway by removing Lrp5/6 co-receptors from the plasma membrane (He et al., 2004) (Figure 2A). This study indicated that activation of Dkk1 expression at the end of gastrulation leads to a marked decrease in the expression of NPB markers *pax3a* and *zic3* (Garnett et al., 2012). Through a series of elegant experiments, *cis*-regulatory elements of *pax3a* and *zic3* were identified containing putative TCF/LEF binding sites. One enhancer of *pax3a* (IR1) contained six putative TCF/LEF binding sites, and mutating this enhancer reduced the expression of *pax3a* in the NPB. In addition, they identified two enhancers of *zic3* (E1 and E2) that contain putative TCF/LEF binding sites and mutating E2 decreased expression of *zic3* in the NPB (Garnett et al., 2012).

Overall, findings from these studies indicate a crucial role for Wnt/β-catenin signalling in establishing the NPB during gastrulation (by activating expression of *pax3a* and *zic3*) and NC induction (by activating expression of *foxd3* and *sox10*). These studies used heat-shock inducible constructs to inhibit different components of the Wnt/β-catenin signalling pathway at discrete developmental stages. Notably, both studies identified changes in gene expression of NPB specifiers and NC specifiers upon inhibition of Wnt/β-catenin signalling. This is likely to reflect an ongoing role of Wnt/β-catenin signalling during gastrulation to specify the NPB and subsequently induce the NC within the NPB.

Although only a few genes were identified in the zebrafish NPB and NC directly regulated by Wnt/β-catenin signalling, there are likely to be many other NPB and NC specifiers regulated by Wnt/β-catenin signalling. A recent study in chick embryos examined the nuclear architecture of the cells expressing the pan-NC marker Pax7 using chromatin conformation capture. Strikingly, a map of active enhancers in NCCs during induction stages shows that the most highly enriched motif in their enhancer map were TCF/LEF-binding sites (Azambuja and Simoes-Costa, 2021a). This analysis led the authors to rethink the NC GRN and proposed that the GRN has a hub-and-spoke architecture whereby Wnt/β-catenin signalling is connected to multiple components through these signal-responsive regulatory elements (Azambuja and Simoes-Costa, 2021a). Further in-depth analysis of *cis-*regulatory elements of a direct Wnt target gene in the NC revealed an intricate regulatory system with inputs from multiple upstream signalling pathways and positive and repressive elements that, as a combination, finely tune the expression of NC specifier genes (Azambuja and Simoes-Costa, 2021a, b). Therefore, it is probable that Wnt/β-catenin signalling has a role both in the initial activation of the GRN and subsequently in maintaining and fine-tuning expression of intrinsic factors through regulation of both positive and negative gene regulatory elements.

## Dickkopf Proteins in Neural Crest Induction

The activation of the NC GRN by the Wnt/β-catenin signalling pathway is also regulated by the activity of its antagonists. One member of the Dkk family of secreted Wnt antagonists, Dkk1, was characterised as a potent inhibitor of Wnt/β-catenin signalling from studies in *Xenopus* and mouse embryos (Figure 2). Overexpression of Dkk1 resulted in anteriorized embryos due to increased inhibition of Wnt/β-catenin signalling that induces posterior neural fates (Glinka et al., 1998; Mukhopadhyay et al., 2001). Dkk1 is expressed in the anterior prechordal mesoderm, where it functions to promote anterior neural fates in the neural ectoderm as well as modulating Wnt/β-catenin signalling in the NPB during NC induction (Glinka et al., 1998; Caneparo et al., 2007; Carmona-Fontaine et al., 2007). Consistent with its function as an inhibitor of the Wnt/β-catenin signalling pathway, loss-of-function of Dkk1 in *Xenopus* and mouse embryos resulted in ectopic expression of NPB specifiers and NC specifiers in the anterior neural fold. Therefore, expression of this Wnt/β-catenin signalling antagonist inhibits NC induction and specification in the anterior ectoderm (Carmona-Fontaine et al., 2007).

Despite the well-documented activity of Dkk1 as a negative regulator of Wnt/β-catenin signalling, studies on the role of Dkk2 suggest that this secreted factor can positively and negatively regulate Wnt/β-catenin signalling in a context-dependent manner. In *Xenopus* embryos, Dkk2 overexpression phenotypes are characterised by microcephaly, similar to phenotypes of embryos with ectopic expression of Wnt8 (Wu et al., 2000). In a study of the role of Dkk2 in NC development, MO-knockdown in *Xenopus* embryos resulted in reduced expression of NC specifiers, but not NPB specifiers or mesodermal genes. This suggests that Dkk2 functions during NC induction to exclusively promote the expression of NC specifiers (Devotta et al., 2018). Furthermore, in animal caps exposed to Wnt8 and BMP antagonist (Noggin) to induce NC, knockdown of Dkk2 resulted in depletion of NC specifiers. This is indicative of a requirement for Dkk2 in Wnt/β-catenin signal transduction during NC induction. Consistent with these findings, Dkk2 is expressed in the posterior of the embryo, unlike Dkk1. Furthermore, rescue of NC specifier expression in Dkk2 knockdown embryos was tested using the Gsk3β chemical inhibitor, BIO, to stimulate Wnt/β-catenin signalling (Figure 2A). Intriguingly, they found that this stimulation of Wnt/β-catenin signalling did not restore normal expression levels of the NC specifier *sox10*. The authors, therefore, speculate that Dkk2 functions to positively regulate Wnt/β-catenin signalling independently of Gsk3β (Devotta et al., 2018). An alternative mechanism by which Dkk2 promotes β-catenin-dependent gene transcription in the NC has not yet been identified.

## Further Levels of Wnt/β-Catenin Signalling and NC Induction

It is essential to recognise that the NC is exposed to many extracellular signalling inputs during induction stages. However, it seems that many are involved in regulating directly or indirectly the Wnt/β-catenin signalling pathway. Studies on a DEAD/H-box RNA helicase, DDX3, in *Xenopus* provided insight into the interplay of signalling pathways in the NC. In the first study on DDX3 function, this RNA helicase was shown to activate Wnt/β-catenin signalling by stimulating CK1 phosphorylation of Dvl, resulting in increased formation of Wnt signalosomes and increased expression of downstream genes (Cruciat et al., 2013). More recently, an in-depth study of DDX3 in *Xenopus* NC development demonstrated that activation of this RNA helicase stabilises β-catenin, activating the expression of NPB and NC specifiers. Rather than directly modulating elements of the Wnt/β-catenin pathway, the authors found that DDX3 regulates the serine/threonine kinase, AKT, in an RNA helicase-dependent manner. In the NC, DDX3 RNA helicase activity stimulates AKT, which phosphorylates and inhibits Gsk3β, resulting in the accumulation of β-catenin (Perfetto et al., 2021). AKT is also regulated upstream by phosphatidylinositol 3-kinase (PI3K) implicated in NC induction (Ciarlo et al., 2017). Therefore, there is evidence of an interplay between the PI3K-AKT and Wnt/β-catenin signalling pathways, which has downstream effects on the NC GRN. Furthermore, there is evidence of an interplay between BMP and Wnt/β-catenin signalling pathways reported in *Xenopus*. Mesodermal expression of *wnt8* is dependent on BMP signalling (Hoppler and Moon, 1998). More recently, BMP responsive elements were identified upstream of the transcriptional start site of *wnt8*. The transcriptional activator of the BMP signalling pathway, Smad1, was able to bind these *cis*-regulatory elements in the presence of a scaffold protein, Fhl3 (Alkobtawi et al., 2021). A further interaction has been reported on the level of the secreted BMP antagonist Gremlin (Pegge et al., 2020), which interacts with heparan sulfate proteoglycans (HSPGs), a family of cell surface macromolecules that regulate Wnt distribution and signalling (reviewed in Routledge and Scholpp, 2019). This coordination of multiple signalling pathways is often overlooked in NC studies which focus on the function of signalling pathways separately. These studies provide evidence of other signalling pathways that modulate Wnt/β-catenin signalling during NC induction.

**FIGURE 4:**
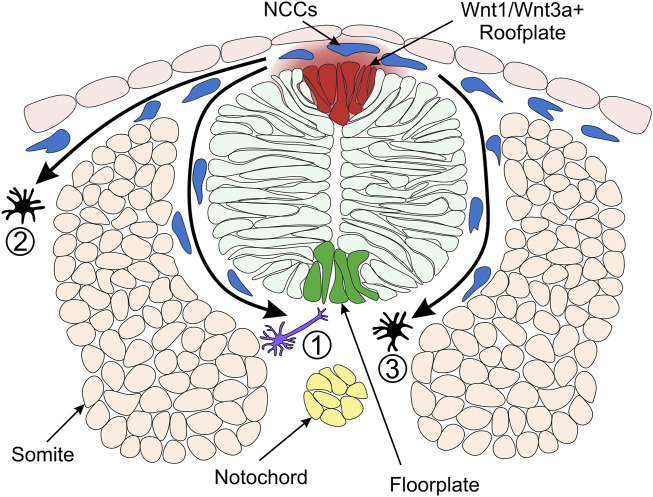
Model of Neural Crest Fate Specification in Zebrafish. Wnt/β-catenin signalling ligands such as Wnt1 and Wnt3a are expressed in the zebrafish dorsal neural tube and bias the fate of NCCs (neural crest cells). The trunk NCCs begin to migrate through the ventrolateral pathway between the neural tube and somites (1). These cells receive a low level of Wnt/β-catenin signalling and are biased towards a neuronal cell fate. For example, the early migrating NCCs taking the ventrolateral pathway give rise to dorsal root ganglia of the peripheral nervous system (Artinger and Bronner-Fraser, 1992). NCCs originating from the dorsal-medial region of the neural plate border are exposed to a high level of Wnt ligands and form pigment cells. These cells of the melanocytic lineage migrate through the dorsolateral pathway between the ectoderm and somite (2) (Henion and Weston, 1997). In a second migratory wave, pigment cells can also take the ventrolateral route (3). Cross-section of zebrafish neural tube **(dorsal to the top)** illustrating overlying epidermis, neural plate border region, neural tube, somites (simplified as the dorsal muscle block) and notochord on the dorsoventral axis.

Given the precise requirement of Wnt/β-catenin signalling during NC induction, it is not surprising that several studies have implicated specific Wnt/β-catenin ligands in the process. Wnt8a was implicated in NPB specification and NC induction through Morpholino-based knockdown studies (Garnett et al., 2012; Lewis et al., 2004). However, further Wnt/β-catenin ligands originating from the neural ectoderm have been implicated in NC induction. In mice, Wnt1, Wnt3 and Wnt3a are expressed in the dorsal roof plate of the neural tube during the stages of NC induction (Parr et al., 1993). Furthermore, mouse Wnt1 and Wnt3a double mutants displayed defects in NC formation, consistent with the role of Wnt/β-catenin signalling in NC induction (Ikeya et al., 1997). Similarly, zebrafish Wnt1 and Wnt3a are expressed in the dorsal neural keel adjacent to the NPB (Dorsky et al., 1998) (Figure 4). Wnt3 is also expressed in the zebrafish dorsal roof plate; however, whether Wnt3 has a functional role in NC development has not yet been explored (Teh et al., 2015). Strikingly, it was demonstrated that the murine mutants of Wnt1 and Wnt3a not only displayed a reduction in the size of NC clones, which would be indicative of a function in NC induction, but they also altered the balance of NCC derivatives. This led to the perception that Wnt/β-catenin signalling has a continued role in NC development following NC induction (Ikeya et al., 1997).

### NC Fate Specification

Analysis of the role of Wnt1 and Wnt3a signalling in mouse and zebrafish NC shows that fates of NCCs are biased by Wnt/β-catenin signalling (Dorsky et al., 1998; Ikeya et al., 1997). In a study of cranial NC, fluorescein-dextran cell labelling suggested that NCCs located ventrolaterally furthest away from the origin of Wnt/β-catenin signals in the neural keel are destined to become neuronal cells (Dorsky et al., 1998) (Figure 4). On the other hand, NCCs that form pigment cell derivatives are located in the medial domain of the NC, adjacent to the source of Wnt1 and Wnt3a ligands in the neural keel (Schilling and Kimmel, 1994) (Figure 4). Furthermore, overexpression of β-catenin in NCCs in the lateral domain resulted in pigment cell generation instead of neuronal cells. Conversely, injection of a dominant-negative Wnt1 or a mutated form of TCF3 into medial NCCs generated neuronal cell types instead of pigment cells (Dorsky et al., 1998). These early experiments established the long-standing model whereby NCCs receiving a high level of Wnt/β-catenin signalling are biased towards pigment cell fates.

In contrast, NCCs that receive low levels of Wnt form neuronal cell derivatives. These findings demonstrate a requirement for Wnt/β-catenin signalling in the specification of NC pigment cell derivatives over neuronal derivatives. How can NCCs acquire different fates if all of them originate from one cell population adjacent to the same signalling source, the Wnt1/Wnt3a^+^ roof plate? There are two hypotheses explaining this paradox. The NCCs specified to neuronal lineages could delaminate and migrate away from the dorsal neural tube earlier than NCCs specified as melanocytes. Such a temporal difference in migration of NC derivatives could result in different periods of exposure to Wnt ligands, with melanophores exposed to Wnt for a longer duration than neuronal cells (Figure 4) (Dorsky et al., 1998; Nitzan et al., 2013). Indeed, the transition from pre-migratory to migratory NCCs is facilitated by Cadherin2 (Ahsan et al., 2019; Scarpa et al., 2015). By blocking Cadherin2 function, pre-migratory NCCs accumulate at the dorsal midline, adjacent to the Wnt1/Wnt3a^+^ roof plate (Figure 4) and start to express the pigment cell marker *microphthalmia-associated transcription factor a* (*mitfa*) (Piloto and Schilling, 2010; Tuttle et al., 2014). However, employing a high-resolution method of detecting RNA transcripts in whole-mount embryos suggested that these NCCs may not be fate-restricted to the pigment cell lineage at this stage (Tatarakis et al., 2020). Therefore, despite the long-standing model of NC fate specification whereby Wnt1/Wnt3a ligands originating in the dorsal neural tube promote pigment cell fates over neuronal cell fates, the underlying mechanisms of the Wnt morphogen gradient and the timing of specification is not fully understood.

## Wnt/β-Catenin Signalling and Pigment Cell (Chromatophore) Specification

The specification of the pigment cell lineages of the NC has been extensively studied due to their distinctive colour and morphology that enables easy identification of mutants in genetic screens (Kelsh et al., 1996; Lamoreux, 2010). Mammals only have one type of pigment cell, melanocytes, whereas zebrafish have three types; melanocytes, xanthophores and iridophores (Fujii, 1993; Rawls et al., 2001; Schartl et al., 2016). Of these three cell derivatives, the GRN that specifies the melanocyte cell lineage has been best characterised. The NC transcription factor *sox10* is required to specify all three pigment cell derivatives (Kelsh and Eisen, 2000; Dutton et al., 2001). *Sox10* transcription factor is an NC specifier, which is first expressed in pre-migratory NCCs. *sox10* (also known as *colourless*) mutants have defects in all NC derivatives except for ectomesenchymal lineages (Kelsh and Eisen, 2000; Dutton et al., 2001). A similar phenotype was also shown in *sox10* mutant mice (Kapur, 1999). *sox10* expression is transiently maintained in NCCs during migration before being switched off in all lineages apart from iridophores and glia (Dutton et al., 2001). It has been suggested that *sox10* functions in the fate specification of NC derivatives from multipotent progenitors, e.g., sensory neurons (Carney et al., 2006).

In melanocyte specification, Sox10 works in conjunction with Wnt/β-catenin signalling to activate and maintain the expression of the melanocyte master regulator, *mitfa* (Hodgkinson et al., 1993; Opdecamp et al., 1997; Lister et al., 1999; Elworthy et al., 2003; Vibert et al., 2017). Interactions between SOX family transcription factors and Wnt/β-catenin signalling have been widely reported in many systems (Kormish et al., 2010). Recently a study using human pluripotent stem cells showed that two SOX factors, SOX2 and SOX17, directly bind β-catenin and this protein-protein interaction results in the recruitment of β-catenin to lineage-specific regulatory elements in both the presence and absence of TCF/LEF (Mukherjee et al., 2021). These findings lead us to speculate on potential interactions between Sox10 and β-catenin in NCCs, which could mediate a lineage-specific Wnt-responsive transcriptional program.

Similarly to *pax3a* and *zic3*, *mitfa* has *cis*-regulatory elements that contain LEF-binding sites enabling regulation of *mitfa* expression by Wnt/β-catenin signalling, and a different regulatory element containing Sox10-binding sites (Dorsky et al., 1998; Dorsky et al., 2000; Takeda et al., 2000; Jin et al., 2001; Elworthy et al., 2003; Hou et al., 2006). *mitfa* expression is required for the process of melanogenesis and melanocyte cell survival and proliferation (Lister et al., 1999). However, it has been shown that *mitfa* is transiently expressed in all NCCs even though only a subpopulation of NCCs form melanocytes (Curran et al., 2010). Therefore, maintaining *mitfa* expression is crucial for establishing the melanocyte cell lineage (Nikaido et al., 2021).

Experiments with BIO, the chemical inhibitor of Gsk3β, helped further unravel the link between Wnt/β-catenin signalling and melanocyte specification (Vibert et al., 2017). Incubation with this compound stabilises β-catenin to activate Wnt/β-catenin dependent gene expression (Figure 2A). Indeed, zebrafish embryos incubated in BIO at 15–30 hours post-fertilisation (hpf) increased melanocyte specification due to enhanced Wnt/β-catenin signalling. On the other hand, BIO treatment at 24–72 hpf, when *sox10* expression is no longer detected in melanocytes, showed no changes in melanocyte number. However, the melanocyte morphology was affected (Vibert et al., 2017). Therefore, a two-stage model was proposed: At 15–30 hpf, Sox10-mediated melanocyte specification needs Wnt/β-catenin signalling, and at 24–72 hpf, the maintenance of melanocytes requires Wnt/β-catenin signalling together with *mitfa* expression.

One evident bottleneck in our understanding of the specification of these pigment cell lineages is the lack of identification of the Frizzled receptors involved in melanocyte specification. Characterising expression patterns and performing Morpholino-mediated knockdowns of numerous Frizzled genes was used to assess Frizzled receptors as candidates for NC development and pigment cell specification in zebrafish (Nikaido et al., 2013). This analysis, however, proved ineffective; none of the assessed Frizzled receptors was expressed in NCCs or melanocytes, and no pigment cell defects were detected in the knockdowns explored (Nikaido et al., 2013). Thus, the possibility remains that other Frizzled members, not included in the study, may be required in the NC. In addition, other higher-resolution methods to detect expression localisation, such as Nanostring, could provide insight into this elusive receptor repertoire of NCCs (Petratou et al., 2018). Alternatively, it has been suggested that Wnt co-receptors can signal without the need for Frizzled receptors (Brinkmann et al., 2016).

## Models of NC Specification

The mechanisms regulating NC specification, whereby NCCs are fate-restricted to individual cell types, remain controversial. NC fate decisions are imposed by environmental signalling cues, and it was assumed that this results in fully multipotent NCCs becoming determined as unipotent cells of specific fates. Two explanatory models were proposed in the late 20th century. The direct fate restriction (DFR) model was proposed based on single-cell labelling studies in the chick dorsal neural tube and envisaged fully multipotent NCCs as directly adopting single fates; this model was supported by work using rat NC stem cells that identified key extracellular signals that instruct NCCs to adopt an individual fate from multiple options (Bronner-Fraser and Fraser, 1988; Bronner-Fraser and Fraser, 1989; Fraser and Bronner-Fraser, 1991; Stemple and Anderson, 1992).

The second model, and nowadays the prevailing one, has been the progressive fate restriction (PFR) hypothesis. The PFR hypothesis proposes that multipotent NC progenitor fates adopt specific fates through a series of partially restricted intermediate progenitors, each with limited but distinct potencies (Sieber-Blum and Cohen, 1980; Weston, 1991; Calloni et al., 2009). Single-cell profiling of mouse NC has supported this model by tracking pre-migratory NCCs through successive fate restrictions towards neural and ectomesenchymal fates (Soldatov et al., 2019). Similarly, single-cell RNA analysis of NC-derived cells taken from various stages of embryonic, larval, juvenile and adult fish have each identified putative pigment cell progenitors (Lencer et al., 2021; Howard IV et al., 2021; Saunders et al., 2021), although in each case the markers used to identify these progenitors does not include all the genetically best-characterised genes with known functional roles in pigment cell fate choice so that the exact identities of these cells and the relationship between the cells described in each study merits further detailed investigation. Thus, the PFR hypothesis has become dominant over the alternative DFR model.

However, a direct test of the PFR model for zebrafish pigment cell development, using sensitive NanoString single-cell profiling of NCCs throughout embryonic development, unexpectedly failed to identify the predicted tripotent (chromatoblasts) and bipotent (melanoiridoblast) progenitors (Nikaido et al., 2021). Furthermore, the same study revealed broad potency, for pigment *and* neural fates, for early NCCs expressing *leukocyte tyrosine kinase*, *ltk*, previously hypothesised to be a chromatoblast marker. The authors proposed an alternative model of NC fate restriction, known as the cyclical fate restriction (CFR) hypothesis, which proposes a more dynamic view of NC specification than either the PFR or DFR models. In the PFR model, the transition from one progenitor state to another of more restricted potency (e.g., from chromatoblast to melanoiridoblast) is associated with loss of potential for one or more fates. In contrast, the CFR hypothesis proposes that NC-derived Highly Multipotent Progenitors cycle through different sub-states, each primed towards a particular cell fate; however, because these cells cycle through sub-states primed for all fates, in turn, they retain multipotency, even although in a “snap-shot” view they appear fate-specified. It is proposed that the priming is reflected in fluctuating expression levels of fate-specification receptors and key fate-specific transcription factors such as Mitfa. The CFR hypothesis suggests that adopting a specific fate occurs when the primed sub-state is exposed to sufficient levels and duration of the fate-specification ligand, driving the differentiation of a specific fate (Kelsh et al., 2021). Although the CFR hypothesis is, at this point, somewhat speculative, it is supported by several observations and by theoretical modelling studies (Farjami et al., 2021). For example, the *ltk*, encoding a receptor tyrosine kinase, is crucial for iridophore specification but shows heterogeneous expression in premigratory NCCs (Lopes et al., 2008). The CFR model also helps to explain recent observations of a group of NC progenitors that simultaneously express factors involved in the specification of all pigment cell lineages – *ltk* and *tfec* (iridophores (Lopes et al., 2008; Petratou et al., 2021), *mitfa* (melanocytes (Lister et al., 1999)) and *pax7* (xanthophores (Minchin and Hughes, 2008)), but also factors crucial for neural fate specification (Nikaido et al., 2021). One important, but underappreciated, implication of all of these observations is that fate restriction to a specific fate depends as much on the repression of fate-specific transcription factors and receptors specifying other fates, as well as the upregulation and maintenance of those for the selected fate (Petratou et al., 2018). Thus, the upregulation of Ltk and Tfec, as well as downregulation of, for example, a Frizzled receptor and Mitfa, all in response to exposure to ALKALs (ligands of Ltk) would result in an iridophore fate. In contrast, upregulation of Frizzled and Mitfa, following exposure to Wnt/β-catenin ligands and downregulation of Ltk and Tfec (and others) would drive the cell towards a melanocyte fate (Kelsh et al., 2021). It is conceivable that Wnt signalling plays a key role in the entry and exit from the NC-HMP cycling progenitor state and may act alongside other signals to initiate entry into the cycling state and to allow differentiation of each derivative fate from the cells in the cycling state (Farjami et al., 2021). The CFR model shares similarities with the so-called “phase-stage model”, which has been proposed for embryonic stem (ES) cell fate restrictions following the observation that an ES cell can occupy a state, known as the phase stage when it can “explore” several potential states (distinguished by oscillating levels of Nanog; Miyanari and Torres-Padilla, 2012) over time (Garcia-Ojalvo and Martinez Arias, 2012). In conclusion, the CFR offers a novel and dynamic framework explaining the previous data supporting either the DFR or the PFR model. Distinguishing these models will require sensitive assessment of the broadest range of markers of each cell-fate, especially those known to drive fate decisions, and development of tools for highly sensitive detection of the dynamic expression of transcription factors and fate-specification receptors in living embryos.

## Concluding Remarks and Future Perspectives

The vertebrate novelty of the NC continues to fascinate the scientific community. NC research has benefitted from applying multidisciplinary techniques, from the cell lineage tracing experiments pioneered by Nicole Le Douarin to the single-cell sequencing technologies that continue to uncover the NC GRN. In this review, we have discussed the current knowledge of the role of Wnt/β-catenin signalling in NC induction and specification. It is well-established that β-catenin-dependent Wnt signalling is required for NC induction and specification, particularly during pigment cell specification. Although some canonical Wnt ligand candidates have been implicated in NC development, there remains a significant lack of understanding of the mechanism of Wnt ligand transport during NC development. Localising these ligands and clarifying their mechanisms of transport would allow refinement of our knowledge of when and how Wnt/β-catenin signalling influences NC development at each of the stages outlined above.

Wnt morphogens are hydrophobic due to post-translational modification with lipid moieties during intracellular trafficking (Willert et al., 2003; Takada et al., 2006). It is, therefore, improbable that Wnt ligands are secreted by cells and diffuse freely through extracellular space (reviewed in Routledge and Scholpp, 2019). As a result, several alternative mechanisms of long-range Wnt transport have been explored, including specialised signalling filopodia, known as cytonemes (Stanganello et al., 2015; Brunt et al., 2021). Although there is strong evidence for canonical Wnt expression in the dorsal roof plate of the zebrafish neural tube during NC induction and specification stages, there is no published research on the ligand transport mechanisms regulating this process. Therefore, high-resolution imaging could be utilised to visualise the mechanism of Wnt transport during NC development. Zebrafish is the ideal model organism for this research, given the powerful transgenic tools and superb *in vivo* imaging due to the embryos’ transparency.

Furthermore, studying the function of the Wnt/β-catenin signalling pathway during NC development has proven challenging due to its necessity during early embryonic development, in gastrulation for AP patterning, and neural plate patterning. In this review, we have highlighted studies in which authors have provided temporal control to the modulation of the Wnt/β-catenin signalling pathway, for example, using the heat-shock promoter or incubation of chemical inhibitors at different developmental stages (Lewis et al., 2004; Garnett et al., 2012; Vibert et al., 2017). However, studies have also relied on whole organism knockouts and MO-based knockdowns to study the impact of specific Wnt ligand candidates on NC development. Such a strategy is not ideal as Wnt/β-catenin signalling is vital in the early embryo and successive stages of NC development. Therefore, it is unclear whether the phenotypes observed in the NC result from inhibition of Wnt/β-catenin signalling during NC stages of development or whether researchers observe downstream effects from inhibition of Wnt/β-catenin signalling earlier in development. As an alternative, we propose using conditional gene knockouts that can be spatially and temporally controlled in the developing embryo. Such a strategy could enable the knockout of essential components of the Wnt/β-catenin signalling pathway only in NCCs during NC induction or specification stages. Indeed, conditional knockout lines using CRISPR/Cas9 technology are appearing in the literature. For example, the Cre/LoxP system has been introduced to control the expression of Cas9 and gRNAs in zebrafish (Hans et al., 2021). Further research will be required to compare the relative efficiency and specificity of this novel conditional knockout system. However, it is undeniable that these new knockout strategies will be invaluable to the future of NC research.
